# Treatment Strategies Used in Treating Myelofibrosis: State of the Art

**DOI:** 10.3390/hematolrep16040067

**Published:** 2024-10-30

**Authors:** Massimo Martino, Martina Pitea, Annalisa Sgarlata, Ilaria Maria Delfino, Francesca Cogliandro, Anna Scopelliti, Violetta Marafioti, Simona Polimeni, Gaetana Porto, Giorgia Policastro, Giovanna Utano, Maria Pellicano, Giovanni Leanza, Caterina Alati

**Affiliations:** 1 Hematology and Stem Cell Transplantation and Cellular Therapies Unit (CTMO), Department of Hemato-Oncology and Radiotherapy, Grande Ospedale Metropolitano “Bianchi-Melacrino-Morelli”, 89133 Reggio Calabria, Italy; dr.massimomartino@gmail.com (M.M.); annalisa.sgarlata@ospedalerc.it (A.S.); ilariamaria.delfino@ospedalerc.it (I.M.D.); francesca.cogliandro@ospedalerc.it (F.C.); anna.scopelliti@ospedalerc.it (A.S.); violetta.marafioti@ospedalerc.it (V.M.); polimenisimona99@gmail.com (S.P.); porto.tania25@gmail.com (G.P.); giorgia.policastro00@gmail.com (G.P.); giovanna.utano@ospedalerc.it (G.U.); maria.pellicano@ospedalerc.it (M.P.); caterina.alati@ospedalerc.it (C.A.); 2 Stem Cell Transplant Program CIC587, 89133 Reggio Calabria, Italy; 3 Pharmacy Unit, Grande Ospedale Metropolitano ‘Bianchi-Melacrino-Morelli’, 89128 Reggio Calabria, Italy; ranza86@gmail.com

**Keywords:** myelofibrosis, JAK inhibitors, ruxolitinib, fedratinib, pacritinib, momelotinib, allogenic stem cell transplantation

## Abstract

Background: Current drug therapy for myelofibrosis does not alter the natural course of the disease or prolong survival, and allogeneic stem cell transplantation is the only curative treatment modality. For over a decade, the Janus kinase (JAK) inhibitor ruxolitinib has been the standard of care. More recently, newer-generation JAK inhibitors have joined the ranks of accepted treatment options. Objectives: The primary goal of treatment is to reduce spleen size and minimize disease-related symptoms. Prognostic scoring systems are used to designate patients as being at lower or higher risk. For transplant-eligible patients, transplant is offered to those with a bridge of a JAK inhibitor; patients who are not eligible for transplant are usually offered long-term therapy with a JAK inhibitor. Limited disease-modifying activity, dose-limiting cytopenias, and other adverse effects have contributed to discontinuation of JAK inhibitor treatment. Conclusions: Novel JAK inhibitors and combination approaches are currently being explored to overcome these shortcomings. Further research will be essential to establish optimal therapeutic approaches in first-line and subsequent treatments.

## 1. Introduction

Primary myelofibrosis (MF) is characterized by an abnormal proliferation of megakaryocytes and granulocytes in the bone marrow, with a polyclonal increase in fibroblasts and fibrotic stages that drive secondary reticulin and collagen marrow fibrosis, extramedullary hematopoiesis, and osteosclerosis [1]. While it is easy to diagnose MF compared with chronic myeloid leukemia, the differential diagnosis is more complex for polycythemia vera (PV) and essential thrombocythemia (ET) (Table 1) [2,3,4]. The annual incidence is 0.22–0.99 cases per 100,000, with a difference between North America/USA (0.33–0.46 cases per 100,000) and Europe (0.1–1 case per 100,000) [5,6,7,8]. MF is more common in men (0.44–0.59 cases per 100,000) than in women (0.24–0.30 cases per 100,000), with a median age at diagnosis of 65 years. Median survival is between 2.3 and 11.3 years and dependent on risk scores [9]. Five-year survival for higher-risk patients is as low as 18% in the US and 35% in Europe [10], and primary MF is associated with higher rates of transformation to MDS or AML and mortality [11,12].

Current drug therapy for MF does not modify the disease’s natural history or prolong survival [13,14], and allogeneic stem cell transplantation (allo-SCT) represents the only curative treatment modality [15].

## 2. Diagnostic Criteria and Risk Stratification

Diagnostic criteria for primary MF require that patients have all three major criteria and ≥1 minor criteria (Table 2) [16]. JAK2, MPL, and CALR mutations are considered driver events, but these mutations are also prevalent in PV and ET. Other mutations such as ASXL1, DNMT3A, and TET2 coexist in approximately half of patients. Mutations affecting splicing regulators of chromatin structure, epigenetic functions, and cellular signaling, such as ZRSR2, EZH2, IDH1, IDH2, CBL, KRAS, NRAS, TP53, SRSF2, SF3B1, U2AF1, and STAG2, are less frequent [17]. The 2016 WHO classification system distinguishes ‘prefibrotic’ from ‘overtly fibrotic’ MF, and about 15% of patients with PV or ET could progress into a PMF-like phenotype during their clinical course. ASXL1, U2AF1-Q157, and SRSF2 mutations are more common in MF and advanced disease, and some correlate with poorer survival. RAS/CBL mutations predict resistance to ruxolitinib (RUXO) therapy. Very high-risk abnormalities include +21, +19, 12p-, −7, 11q-, inv (3), and i (17q).

The International Prognostic Scoring System (IPSS), Dynamic International Prognostic Scoring System (DIPSS), and DIPSS plus (Table 3) represent the classic prognostic models for MF [18,19,20]. The IPSS classifies the disease at diagnosis and includes five independent predictors of inferior survival: hemoglobin < 10 g/dL, age  >  65 years, leukocyte count > 25 × 10^9^/L, the presence of constitutional symptoms, and circulating blasts ≥ 1%. The presence of 0, 1, 2, and  ≥ 3 adverse factors define low, intermediate-1, intermediate-2, and high-risk disease. The DIPSS is characterized by the same prognostic variables used in the IPSS, applied any time during the disease course. The DIPSS assigns two adverse points for hemoglobin < 10 g/dL, and risk categorization is accordingly modified: low (0 adverse points), intermediate-1 (1 or 2 points), intermediate-2 (3 or 4 points), and high (5 or 6 points). Incorporating three additional independent risk factors (platelet count < 100 × 10^9^/L, red cell transfusion needs, and unfavorable karyotype) led to the development of the so-called DIPSS-plus. The four DIPSS-plus risk categories based on the eight risk factors are low (no risk factors), intermediate-1 (one risk factor), intermediate-2 (two or three risk factors), and high (four or more risk factors).

Two new prognostic systems were recently introduced: a genetically inspired prognostic scoring system (GIPSS) and a mutation- and karyotype-enhanced international prognostic scoring system (MIPSS70+ version 2.0) [21]. The GIPSS is based on any abnormal karyotype other than normal or sole abnormalities of 13q-, 20q-, +9, chr.1 translocation/duplication, -Y, or a sex chromosome abnormality other than -Y, and any mutation in EZH2, SRSF2, ASXL1, or IDH1/2. The MIPSSv2 includes clinical risk factors, such as peripheral blast > 2%, Hb < 10 g/dL, and constitutional symptoms.

## 3. First-Line Therapy

The main goals of MF treatment are to reduce spleen volume (SV) and improve clinical symptoms and survival. An observational strategy is reserved exclusively for MIPSSv2 “low” and “very low” risk disease, where the estimated 10-year survival is between 56% and 92%. Treatment with JAK inhibitors can be considered the standard of care without a suitable clinical trial. With multiple JAK inhibitors now available, patient- and disease-specific factors, particularly cytopenias, drive the therapeutic approach. Allo-SCT should be considered at diagnosis and remains the only potentially curative therapy. The transplantation approach is associated with a high risk of mortality and morbidity [22], so it is reserved for patients with a worse prognosis. More details on transplant indications will be discussed in the specific section.

The oral Janus kinase (JAK) 1/JAK2 inhibitor ruxolitinib (RUXO) was initially approved by the US Food and Drug Administration in 2011 for the treatment of patients with intermediate- or high-risk MF, including primary MF, post-polycythemia vera MF, and post-essential thrombocythemia MF, based on efficacy and safety findings from the randomized, controlled, phase 3 COMFORT trials [23,24]. Over a decade later, RUXO continues to be the standard of care in higher-risk MF, and dose optimization and management remain crucial for safely maximizing clinical benefits. In the COMFORT-I trial [23], 41.9% of patients in the RUXO arm achieved the primary endpoint of a ≥35% reduction in SV from baseline at week 24. Most patients who received RUXO had improvements in MF-related symptoms, whereas symptoms worsened in most patients who received placebo (PLAC). Development of anemia did not affect response to RUXO treatment. In the COMFORT-II trial [24], 28% of patients who received RUXO achieved the primary endpoint of a ≥35% reduction in SV at week 48. Symptoms, including loss of appetite, fatigue, dyspnea, pain, and insomnia, improved in patients who received RUXO and worsened in patients who received the best available therapy (BAT).

Real-world data confirmed the safety and efficacy of RUXO, including those with low platelet counts [25]. The five-year update from the COMFORT-I and II trials showed that RUXO was associated with long-lasting reductions in SV, with a median duration of ≥35% SV reduction from baseline being about 3.2 years with maintenance of long-term RUXO therapy [26,27]. Early intervention with RUXO showed that IPSS intermediate-1-risk patients experienced lower toxicities than patients with higher-risk diseases and achieved higher response rates [28,29,30]. In pooled COMFORT-I and II analysis, reductions in SV with RUXO treatment correlated with more prolonged survival [31,32]. In a retrospective study of 284 patients treated with RUXO for ≥1 year, SV response at six months correlated with more prolonged survival [33]. In a real-world analysis of patients with intermediate-to-high-risk MF, the mortality risk was lowest, and OS was the longest among RUXO-exposed patients [34]. The recommended dose of RUXO is based on platelet count [35,36]. Neutropenia, thrombocytopenia, or anemia should be managed with dose reductions, interruption, or transfusions. In the REALISE single-arm phase II study, patients with anemia (Hb < 10 g/dL) received an alternative RUXO dosing regimen [37]. Week 24 SV response was observed in transfusion- and non-transfusion-dependent patients. Current guidelines recommend continuing RUXO near the start of conditioning chemotherapy prior to allo-SCT [38]. Patients who receive treatment with RUXO may need to stop because it is not working, or they cannot tolerate the side effects.

**Figure 1:**
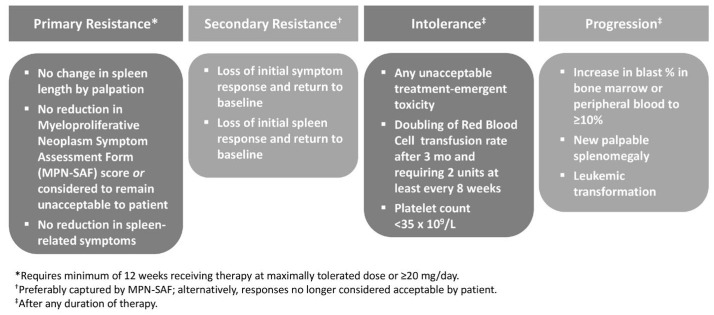
Defining ruxolitinib failure in clinical practice.

Expert consensus has been provided on defining RUXO failure and transitioning to next-line therapy (Figure 1) [39].

Additional JAK inhibitors have been studied for the treatment of MF. Fedratinib (FEDRA) is a JAK2-selective inhibitor, approved in the United States as a therapy for patients with intermediate-2 or high-risk primary or secondary MF after the results of the JAKARTA study [40]. Treatment with FEDRA demonstrated clinically meaningful and statistically significant reductions in SV- and MF-associated symptom burden versus PLAC. In an indirect comparison with RUXO, the efficacy of reducing SV and improving symptomatology was similar. The incidence of thrombocythemia has been lower with FEDRA. However, the drug is characterized by significant gastrointestinal toxicity, with an inhibition of the uptake of thiamine, which has been the cause of neurological toxicity. The phase 3 FREEDOM2 trial results have been presented at ASH 2023. This study compared a treatment approach between FEDRA and BAT with that of RUXO-experienced patients [41]. The primary endpoint of this study was SV reduction at week 35 at the end of cycle 6, and secondary endpoints included symptom response, SV reduction at week 25, durability of SV reduction, and safety. The study showed that patients in the FEDRA cohort experienced significantly higher rates of SV reduction at week 35 (35.8%) compared with BAT (6.0%, *p* < 0.0001). FEDRA-treated patients also had superior SV reduction at week 25 and SV reduction at week 35 and higher symptom response rates (34.1% vs. 16.9%, *p* = 0.0033). No new safety concerns for FEDRA were identified.

Pacritinib (PACRI) is a selective inhibitor of JAK2, JAK2 V617F, and FLT3 and does not inhibit JAK1. Moreover, it has a potential additive anti-inflammatory effect by inhibiting IL-1 receptor-associated kinase 1 and IL–1–related signaling [42]. In the PERSIST-1 [43] and PERSIST-2 [44] phase 3 trials, PACRI has been tested versus BAT for the treatment of MF. The drug induced significant symptoms and sustained SV reduction, even in patients with severe baseline cytopenias or thrombocytopenia. These results led to the approval of PACRI for patients with intermediate- or high-risk primary or secondary MF with platelet counts < 50 × 10^9^/L [45].

Momelotinib (MMB) is an inhibitor of JAK1 and JAK2, an emerging treatment for MF patients [46]. MMB inhibits ACVR1/ALK2, decreases hepcidin production, and ameliorates anemia of chronic disease in rodents, resulting in increased iron availability for erythropoiesis, which can potentially improve anemia [47]. MOMENTUM was a phase 3 study that enrolled adult patients with primary MF or post-polycythemia vera or post-essential thrombocythemia MF. Patients were randomly assigned (2:1) to receive MMB (200 mg orally once per day) plus danazol placebo or danazol (300 mg orally twice per day) plus MMB placebo, stratified by total symptom score (TSS; <22 vs. ≥22), spleen size (<12 cm vs. ≥12 cm), red blood cell or whole blood units transfused in the 8 weeks before randomisation (0 units vs. 1–4 units vs. ≥5 units), and study site. A significantly greater proportion of patients in the momelotinib group reported a 50% or more reduction in TSS than in the danazol group (32 [25%] of 130 vs. six [9%] of 65; proportion difference 16% [95% CI 6–26], *p* = 0·0095) [48]. SIMPLIFY-1 compared MMB versus RUXO in JAK inhibitor-naïve patients and SIMPLIFY-2 MMB vs. BAT in patients with prior RUXO therapy. Transfusion independence response rates were higher for MMB in both SIMPLIFY-1 (67% vs. 49%) and SIMPLIFY-2 (43% vs. 21%). The week 24 transfusion independence response was higher in the MMB arm, regardless of baseline anemia, platelet count, or transfusion status. In SIMPLIFY-1, the 3-year survival in MMB transfusion independence responders was 80% compared with 50% in MMB transfusion independence no responders (HR, 0.30; *p* < 0.0001). Similar results are found for patients with anemia who are week 24 MMB transfusion independence responders. In SIMPLIFY-2, patients randomized to MMB who were transfusion independence responders at week 24 showed a trend toward better OS compared with transfusion independence no responders (HR, 0.52; *p* = 0.0652). Additional research on MMB has also been performed. Investigators aimed to characterize the impact of MMB and comparators (danazol, RUXO) on transfusion burden in JAK inhibitor-naïve and -experienced MF patients. In JAK inhibitor-naïve patients, the mean red blood cell (RBC) transfusion burden per 28 days declined by 0.10 units from baseline in the MMB treatment arm and increased by 0.39 units in the RUXO arm.

Additionally, 87% of those receiving MMB maintained or improved RBC transfusion intensity during treatment compared with baseline versus 54% of those receiving RUXO. In experienced MF, more patients in the MMB arm (35%) required zero units of RBC transfusion compared with the danazol arm (17%). In contrast, the mean RBC transfusion burden per 28 days declined by 0.86 units from baseline in the MMB arm versus 0.28 units in the danazol arm. Across both trials, at least 85% of MMB-treated patients improved or maintained transfusion intensity. The authors concluded that MMB was associated with superior maintenance of RBC transfusion intensity and zero RBC transfusion status versus comparators. Table 4 summaries the main first-line treatment studies.

## 4. Combination Therapies

Exploratory and phase 2 clinical trials have evaluated RUXO in combination with various agents [49]. Pemmaraju et al. evaluated the efficacy of RUXO plus navitoclax (NAVI), an orally available antiapoptotic B-cell lymphoma two protein inhibitor, in patients with MF. In the ongoing phase 3 TRANSFORM-1 trial, patients with JAK inhibitor-naïve MF were randomized to receive NAVI plus RUXO or PLAC plus RUXO [50]. The primary endpoint of an SV reduction of at least 35% was achieved in 63.2% of patients treated with a combination therapy compared with 31.5% of those in the PLAC group. Combination therapy was also associated with a mean change in total symptom score from baseline of −9.7 (versus −11.1 among those receiving placebo, *p* = 0.2852). Most adverse events (AEs) were manageable with dose modification without clinically significant sequelae. Pelabresib (PELA) is a selective oral bromodomain and extra terminal domain inhibitor that is being investigated for MF, with preclinical data supporting the potential combination of this agent with existing therapies, such as JAK/signal transducer and activator of transcription (STAT) inhibitors. MANIFEST-2 is an ongoing phase 3 trial evaluating the combination of PELA and RUXO versus placebo plus RUXO in JAK inhibitor-naïve patients with primary or secondary MF [51]. In Arm 3 of the study, 84 patients were enrolled with a primary endpoint of SV reduction of at least 35% at week 24 and critical secondary endpoints including 50% or more reduction in total symptom score from baseline, the percentage change in total symptom score, and conversion from transfusion dependence to independence, among others. At week 24, PELA/RUXO combination therapy significantly reduced SV (SVR35 66% vs. 35%; *p* < 0.001), double the percentage of patients with both SV reduction of at least 35% and total symptom score response. Patients in the treatment arm experienced fewer anemia, AEs, and higher rates of Hb response. They were less likely to have transfusion requirements than those in the placebo arm [51]. Long-term results from the phase 1 XPORT-MF-034 study of selinexor (SELI) and RUXO therapy in JAK inhibitor-naïve MF patients have been presented at ASH 2023 [52]. SELI is an oral XP01 inhibitor that inhibits multiple MF-relevant pathways, including STAT, ERK, and AKT. This agent may also work synergistically with RUXO. The XPORT-MF-034 trial enrolled 24 patients to receive SELI 40 mg and RUXO 60 mg, with assessments including safety, rate of SVR35, total symptom score, and platelet and Hb levels. Longitudinal clinical biomarker assessments included the percent of variant allele frequency (%VAF) change at week 24 for driver genes and plasma cytokine levels at week 4. Of 13 patients with available data at week 24, 5 patients (38%) had a ≥20% reduction of VAF, 3 of whom had high VAF (>50%) driver mutations at baseline and were characterized as high molecular risk (HMR). This therapeutic combination also caused a rapid and durable decrease in pro-inflammatory MF-relevant cytokines in most treatment groups. The reduction in IL-18 correlated to SVR and TSS response at week 24. The authors concluded that promising biomarker and efficacy data suggest that SELI, in combination with RUXO, has the potential to become a novel, first-line treatment for patients with MF. SELI is intended to be evaluated in combination with RUXO in JAK inhibitor-naïve MF patients in phase 3 of the XPORT-MF-034 clinical trial series, as well as alone in JAK inhibitor-naïve MF patients with moderate thrombocytopenia in the phase 2 XPORT-MF-044 study.

## 5. Disease Progression or Intolerance to Initial Therapy

Table 5 summaries trials investigating possible therapeutic approaches for disease progression or intolerance to initial therapy. Fedratinib (FEDRA) is an oral selective inhibitor of JAK-2 and FMS-like tyrosine kinase three that was previously shown in the JAKARTA2 and FREEDOM studies to confer SV and symptom reduction in MF patients treated with RUXO [53,54]. At ASH 2023, results from the phase 3 FREEDOM2 trial comparing FEDRA to BAT in RUXO-experienced MF patients were presented [41]. The study showed that patients in the FEDRA cohort experienced significantly higher rates of SV reduction of at least 35% (35.8%) compared with BAT (6.0%, *p* < 0.0001) and higher rates of symptom response (34.1% vs. 16.9%, *p* = 0.0033). No new safety concerns for FEDRA were identified. Pacritinib (PACRI) is a JAK2/IRAK1/ACVR1 inhibitor enhancing erythropoiesis and reducing transfusion dependence [55]. PAC203 was a randomized dose-finding study of PACRI in patients with advanced MF who are intolerant or resistant to RUXO [56]. Patients were randomized to PACRI 100 mg once daily, 100 mg twice daily, or 200 mg twice daily. Efficacy was based on ≥35% SV response and ≥50% reduction in the 7-component total symptom score through week 24. Of 161 patients, 73% were intolerant, 76% had become resistant to RUXO, and 50% met both criteria. Severe thrombocytopenia (platelet count < 50 × 10^3^/μL) was present in 44%. SV reduction rates were highest with 200 mg twice per day (100 mg once per day, 0%; 100 mg twice per day, 1.8%; 200 mg twice per day, 9.3%), particularly among patients with baseline platelet counts < 50 × 10^3^/μL (17%; 4 of 24). Although the total symptom score response rate was similar across doses, the median percent reduction in total symptom score suggested a dose-response relationship. Pharmacodynamic and pharmacokinetic modeling based on all available data showed the greatest total symptom score and SV reduction at 400 mg per day compared with lower doses. Common AEs were anemia, gastrointestinal events, and thrombocytopenia. PACRI 400 mg per day showed clinical activity with an acceptable safety profile and was selected as the recommended dose for a phase 3 study in patients with severe thrombocytopenia (<50 × 10^9^/L) [57].

PERSIST-2 looked at patients with platelets of 100,000/μL or less [58]. Patients were given either 400 mg daily or 200 mg of twice daily PACRI vs. patients on BAT, which could also include RUXO, with a coprimary endpoint of SV reduction of 35% or more and reduction of the total symptom score at week 24. In the BAT arm, 45% of patients got low-dose RUXO, 19% had no options, while some patients were getting hydroxyurea [19%], steroids [13%], and even interferons [2%]. These patients had higher-risk diseases; half had Hb less than 10 g/dL, and the median platelet count was around 50,000/μL in most patients in both arms. For the patients who got 200 mg of PACRI twice daily, 18% had an SV reduction of 35% or more vs. 3% from the BAT arm. For patients with less than 50,000/μL platelets specifically, 29% had an SV reduction of 35% or greater vs. 3.1% in the BAT arm, which included those patients on low-dose RUXO. Fifty percent of the total symptom score is considered the regulatory benchmark for symptom improvement, and that was met in 32% of patients given PACRI vs. 14% in the BAT arm. There were anemia responses that looked different from what we saw with RUXO and FEDRA. If we look at RBC transfusions at units per month, it was 1.06 at baseline, and at week 24, it was reduced to 0.67 with PACRI. We were also seeing these improvements in hemoglobin levels and reduced transfusion burden. There is relative stability in the platelet count with PACRI. At week 24, there was a dip of about 15% to 20%, which is very different from the minus 40% to 45% obtained with RUXO.

In a safety analysis, lower bleeding and cardiac event rates were reported in PAC203 vs. PERSIST-2, likely due to enhanced patient selection, monitoring, and dose modification guidelines [59,60]. Moreover, time at risk for AEs varied for PACRI vs. BAT (including RUXO) due to crossover. The risk-adjusted safety profile of PACRI 200 mg BID was comparable to BAT. Rates of bleeding, including in patients with severe thrombocytopenia, were similar for PACRI vs. BAT or RUXO. Rates of fatal events were higher for RUXO than PACRI. New data have emerged from the phase 3 PERSIST-2 clinical trial analysis, which compared PACRI therapy to BAT in cytopenic MF patients. Data from these two trials showed that 19 of 117 patients (16%) in the PACRI treatment arms experienced a hematologic improvement in platelets, 14 of whom had sustained improvement over ≥12 weeks. In contrast, 4 of 77 individuals in the BAT arm (5%) achieved hematologic improvement in platelets. These results did not appear to be explained by changes in SV. However, they may be the result of modulation of the bone marrow microenvironment and thrombopoiesis stemming from the mechanism of action of PACRI as an IRAK1 inhibitor.

## 6. Allo-SCT

Despite the development of JAK inhibitors, the number of MF patients undergoing allo-SCT is steadily increasing, and the procedure remains the only curative treatment for these patients [61]. Over the years, allo-SCT has become safer with reduced non-relapse mortality (NRM), making this procedure more attractive [62]. Biological stratification of the disease and the use of JAK inhibitors result, on the one hand, in the selection of patients most likely to benefit from transplantation and, on the other hand, in symptom and disease control, which allows the patient to arrive earlier and in a better clinical condition for transplantation [30,63].

**Figure 2:**
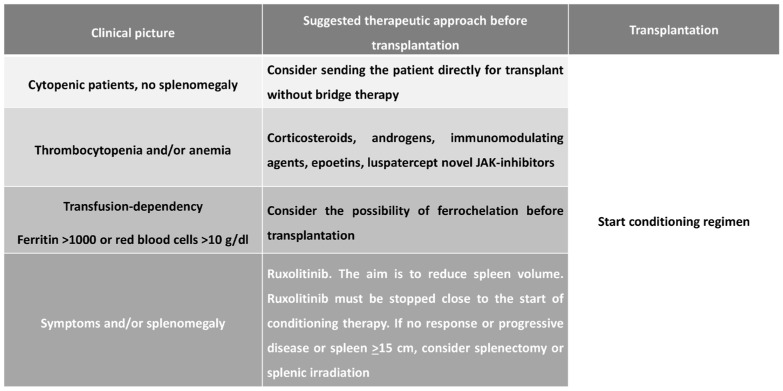
Management of patient candidates for allogeneic stem cells transplantation.

The factors that impact the transplant outcome most are the donor type, stem cell source, conditioning [64,65,66], and the clinical management of MF patients waiting for a transplant [67,68]. In the effort to implement all disease and transplant-specific scores, all MF patients up to 75 years with high-risk disease (as defined as life expectancy lower than five years according to standard MF-oriented prognostic scores) should be considered possible candidates for a transplant. For those patients with low–intermediate-risk MTSS with no significant contraindications, transplant should be pursued as soon as possible; on the contrary, other treatment options should be evaluated in very high high-risk MTSS category or with severe comorbidities, in a high MTSS risk group of patients, where a 5-year NRM of 36% after transplant is expected, an allo-SCT procedure should be chosen on a case-by-case basis considering patient preference and other relevant factors, such as comorbidities, cognitive status, and geriatric assessment. Sibling donors and high stem cell doses should be considered the best approaches [69]. The current EBMT/ELN consensus guidelines suggest tailoring the conditioning regimen intensity based on a patient’s fitness and disease status [70,71]. Therefore, it is crucial to pay particular attention to splenomegaly management before transplant [72] (Figure 2).

## 7. Investigational Non-JAK Inhibitors for MF

Most non-JAK agents tested have shown modest benefit in improving the efficacy of RUXO. Several novel agents such as the activin receptor ligand trap luspatercept, BET inhibitor CPI-0610, telomerase inhibitor imetelstat, and recombinant pentraxin–PRM-151 have shown promising activity. However, they require evaluation in randomized trials to understand the clinical benefit [49]. Drugs that target new molecular pathways (TIM-3, MDM2, aurora kinase, *p*-selectin, TGF-β,) and immune-based strategies (anti-PD-1, allogeneic cord blood regulatory T cells, CALR vaccine) are in early phase trials. Further translational studies to target leukemic stem cells and improvement in trial designs by incorporating control arm and survival endpoints will play a role in MF drug development.

## 8. Expert Opinion

MF is an uncommon disease that belongs to the family of myeloproliferative neoplasms. In 2024, we treat patients with primary or secondary MF the same, without specific guidelines that differentiate them. Moreover, although patients may harbor different driver mutations, such as JAK2, MPL, or CALR, all currently approved therapies are neutral. The main goal of treatment is to reduce SV and minimize MF-related symptoms, thus improving the quality and quantity of life. Clinicians should discuss treatment goals with patients, including the potential use of more aggressive therapeutic options, and that results can be achieved dynamically in the natural course of the disease. Prognostic scoring systems are used to designate patients as having lower- or higher-risk MF. Clinicians focus on symptom management, observation, and management of cytopenias or other disease manifestations for patients with lower-risk disease. Lower-risk patients may benefit from early intervention with a JAK inhibitor and early referral for allo-SCT with curative intent. For patients who are transplant eligible, transplant is offered to those after a bridge of a JAK inhibitor; patients who are not transplant eligible are usually offered long-term therapy with a JAK inhibitor. Patients classified as intermediate-2 or high-risk based on prognostic scoring models often require more immediate therapy.

Currently, the FDA approves four JAK inhibitors for managing patients with MF. RUXO and FEDRA are approved for patients with higher-risk MF, and dosing is not recommended in patients with platelet counts < 50 × 10^9^/L. PACRI is approved for patients with higher-risk MF and a platelet count < 50 × 10^9^/L. PACRI, with its distinctive mechanism of action, may offer a unique survival advantage for MF patients with moderate or severe thrombocytopenia. MMB is approved for patients with higher-risk MF and anemia. Based on these indications and clinical data, the first decision-making point is platelet count. For patients with severe thrombocytopenia or a platelet count < 50 × 10^9^/L, PACRI is preferred, and MMB may be considered. All JAK inhibitors are approved for patients with platelet counts > 50 × 10^9^/L, with RUXO preferred as first-line therapy. Of course, transplant-eligible patients should be referred to a transplant team as early as possible to evaluate their candidacy for a transplant. Although we have four approved JAK inhibitors, they still need to be met. Many patients are primarily refractory to JAK inhibitors, and others have suboptimal responses. Many patients continue to relapse after therapy with short-duration JAK inhibitors. Hopefully, many of the agents under investigation can induce faster and more durable responses that eventually can be disease-modifying. Many agents are under investigation, including those with unique mechanisms of action. At ASH 2023, late-phase clinical trial data were presented for JAK inhibitor—a naive setting, which appears very promising. In TRANSFORM-1, adding navitoclax to ruxolitinib significantly improved the SV reduction rate. However, navitoclax development in MF has currently been stopped.

**Figure 3:**
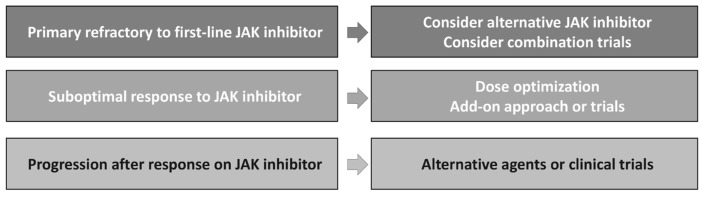
Myelofibrosis: how to choose second-line therapy.

Given the evolution in the treatment landscape for MF, it is essential to consider how to sequence therapy and treat it in the second-line setting (Figure 3) For patients who received a single JAK inhibitor as first-line therapy and develop refractory disease, it is reasonable to offer them an alternative JAK inhibitor. Patients who responded sub optimally to JAK inhibitor monotherapy in the first-line setting may be ideal for combination therapy after dose optimization. In the future, we might start with combinations in most patients, which could change our treatment paradigm significantly. We should consider non-JAK inhibitor agents for patients with multiple JAK inhibitor failures.

RUXO failure remains a question that clinicians face daily as we manage patients. Patients who are primary refractory to a first-line JAK inhibitor are ideal patients to switch therapy. Other patients may respond and then lose that response or can no longer tolerate the drug. Some patients progress beyond MF into the blast phase or acute leukemia and need completely different approaches to their cancer therapy. When selecting subsequent lines of therapy, it is essential to consider the type of resistance or intolerance to treatment a patient experience. This is becoming more relevant as multiple JAK inhibitors are approved beyond RUXO failure and emerging investigational combinations that might be approved in the setting. However, MF treatments continue to have limitations, including treatment failure, dose-limiting cytopenias, and nonhematologic toxicities that are attributed to high rates of treatment discontinuation [73]. Patients with significant symptomatology, including splenomegaly, should be assessed for clinical trials. Without a suitable clinical trial, treatment with a JAK inhibitor is the standard of care in this setting. With multiple JAK inhibitors now available, patient and disease-specific factors are essential in determining the most appropriate therapy. JAK inhibitors remain the foundation of MF therapy, with several trials demonstrating their efficacy. Due to disease evolution or adverse effects, limited disease-modifying activity and dose-limiting cytopenias have contributed to treatment discontinuation. Novel JAK inhibitors and combination approaches are currently being investigated to overcome these shortcomings. Additional research will be paramount to establish the optimal frontline and subsequent treatment approaches in patients with MF.

## Figures and Tables

**Table 1:** Differential diagnosis characteristics among chronic myeloproliferative neoplasms.

Philadelphia Chromosome (BCR/ABL1+)	Chronic Myeloid Leukemia
Philadelphia Chromosome (−)	Essential Thrombocythemia
	Megakaryocyte proliferation (enlarged, mature morphology) JAK2 (~50% of patients), CALR, or MPL mutations Thrombocytosis (platelet count ≥ 450 × 10^9^/L) Progress to AP and BF: low-frequency transformation
	Polycythemia Vera
	Bone marrow trilineage myeloproliferation JAK2 mutation (nearly all patients) Increased Hb, HCT, or RCM Subnormal serum EPO Progress to AP and BF: low-frequency transformation
	Myelofibrosis
	Megakaryocyte proliferation (reticulin/collagen fibrosis) JAK2 (~50% of patients), CALR, or MPL mutations Anemia Leukocytosis Leukoerythroblastosis Splenomegaly Increased serum LDH Leukemic transformation: frequent Leukemia-free survival: shorter in fibrotic than prefibrotic MF

Legend: EPO, erythropoietin; Hb, hemoglobin; HCT, hematocrit; LDH, lactate dehydrogenase; RCM, red cell mass. AP: accelerated phase (10–19% blasts). BP: blastic phase (≥20% blasts). MF: myelofibrosis.

**Table 2:** Primary myelofibrosis: diagnostic criteria.

Major Criteria
Megakaryocyte proliferation and atypia accompanied by reticulin and/or collagen fibrosis or, in the absence of reticulin fibrosis, the megakaryocyte changes accompanied by increased marrow cellularity, granulocytic proliferation and decreased erythropoiesis (i.e., pre-fibrotic primary myelofibrosis) The presence of JAK2, MPL, or CALR mutation or other clonal markers (e.g., EZH2, TET2, IDH1/IDH2, ASXL1, SRSF2, and SF3B1) Not meeting WHO criteria for chronic myeloid leukemia, polycythemia vera, myelodysplastic syndrome, or other myeloid neoplasm
**Minor Criteria**
Anemia not attributed to comorbid conditions, leukocytosis ≥ 11 × 10^9^/L; Palpable splenomegaly LDH increased above the upper limit of normal Leukoerythroblastosis (overt fibrotic primary MF)

**Table 3:** Prognostic Models for Myelofibrosis.

Parameter	International Prognostic Scoring System (IPSS)	Dynamic International Prognostic Scoring System (DIPSS)	Dynamic International Prognostic Scoring System Plus (DIPSS-Plus)
Age > 65 years	Yes (1 point)	Yes (1 point)	Yes *
Hemoglobin < 10 g/dL	Yes (1 point)	Yes (2 points)	Yes *
White Blood Cell > 25 × 10^9^/L	Yes (1 point)	Yes (1 point)	Yes *
Peripheral blood blasts ≥ 1%	Yes (1 point)	Yes (1 point)	Yes *
Platelets < 100 × 10^9^/L	not applicable	not applicable	Yes (1 point)
Red Blood Cell transfusion dependence	not applicable	not applicable	Yes (1 point)
Constitutional symptoms	Yes (1 point)	Yes (1 point)	Yes *
Unfavorable karyotype	not applicable	not applicable	Yes (1 point)
Can be used at any time point	No (only at diagnosis)	Yes	Yes
Risk Group	Median Overall Survival, Years		
Low	11.3	NR	15.4
Intermediate-1	7.9	14.2	6.6
Intermediate-2	4.0	4.0	2.9
High	2.3	1.5	1.3

* 0–3 points for each based on DIPSS risk categories; features not individually weighted.

**Table 4:** First-line therapy.

Study	Patients	Design	Endopoints	Results	Safety
COMFORT-I phase III trial	Primary, post-PV, or post-ET MF; intermediate-2 or high risk; palpable spleen; platelets ≥ 100 × 10^9^/L (*n* = 309)	Ruxolitinib 15 or 20 mg PO twice daily vs. placebo.	Primary endpoint: SVR ≥ 35% at wk 24 (assessed by MRI or CT)	The primary end point was reached in 41.9% of patients in the ruxolitinib group as compared with 0.7% in the placebo group (*p* < 0.001).	Anemia and thrombocytopenia were the most common adverse events, but they rarely led to discontinuation of the drug.
COMFORT-II phase III trial	Primary, post-PV, or post-ET MF; intermediate-2 or high risk; platelets ≥ 100 × 10^9^/L, prior JAK2 inhibitors allowed (*n* = 219)	Ruxolitinib 15 or 20 mg PO twice daily vs BAT. The most common BAT were antineoplastic agents (in 51%)—most frequently hydroxyurea (47%)—and glucocorticoids (16%); a total of 33% of patients received no therapy.	Primary endpoint: SVR ≥ 35% at Wk 48	At week 48, 28% (41/146) of patients randomized to ruxolitinib achieved ≥35% decrease in spleen volume compared with no patients on BAT (*p* < 0.001).	There was no unexpected increased incidence of adverse events with longer exposure.
REALISE Phase II study	Patients with MF and anemia (Hb < 10 g/dL) (*n* = 51)	Alternative ruxolitinib dosing regimen (starting dose 10 mg BID for 12 wk followed by upward titration).	Primary endpoint: proportion of patients achieving ≥ 50% reduction in SL at week 24.	Overall, 70% of patients achieved a ≥50% reduction in palpable spleen length at any time during the study.	The most frequent adverse events leading to dose interruption/adjustment were thrombocytopenia (17.6%) and anemia (11.8%).
JAKARTA double-blind, randomized phase III trial	Adults with primary, post-PV, or post-ET MF; intermediate-2–risk or high-risk status; platelet count ≥ 50 × 10^9^/L; splenomegaly; life expectancy ≥ 6 mo (*n* = 289)	Fedratinib 400 mg PO QD ≥ 6 consecutive 4-wk cycles vs. Fedratinib 500 mg PO QD ≥ 6 consecutive 4-wk cycles vs. placebo PO QD ≥ 6 consecutive 4-wk cycles.	Primary endpoint: spleen response (≥35% reduction in spleen volume vs. BL) at Wk 24 and confirmed 4 wk later.	Spleen Response Fedratinib 400 mg 36.5% Fedratinib 500 mg 40.2% Placebo 1%	Wernicke encephalopathy (ataxia, altered mental status, ophthalmoplegia) occurred in 8 of 608 (1.3%) patients receiving fedratinib in clinical trials.
PERSIST-2 Phase III trial	Adults with PMF or secondary MF with DIPSS intermediate-1 or greater risk disease and moderate to severe thrombocytopenia at baseline (platelets ≤ 100 × 10^9^/L); prior JAK inhibitors allowed (*n* = 311)	Pacritinib 400 mg QD (*n* = 104) vs Pacritinib 200 mg BID (*n* = 107) vs. BAT (*n* = 10). BAT was investigator-determined and included any commercially available therapy (single or combination) or observation only.	Coprimary endpoints: SVR ≥ 35% and ≥ 50% reduction in TSS from baseline to Wk 24 with pooled pacritinib arms vs. BAT.	Spleen and Symptom Response SVR ≥ 35%, Wk 24. ITT Population PAC 200 mg BID = 22%; BAT = 3% Patients With Platelets < 50 × 10^9^/L; PAC 200 mg BID = 2)%; BAT = 3% (*p* = 0.001).	Diarrhea with pacritinib most often occurred during Wk 1–8, was manageable, and resolved within 1–2 wk. Safety outcomes with pacritinib were similar for those with baseline platelets < 50 × 10^9^/L vs. 50–100 × 10^9^/L.
SIMPLIFY-1 Phase III Trial	Adults with primary, post-PV or post-ET MF; int-2 or high risk; platelets 50 × 10^9^/L (*n* = 432). Patients with symptomatic Intermediate-1 risk also included	Momelotinib 200 mg PO daily vs. Ruxolitinib 20 mg PO daily.	Primary endpoint: SVR ≥ 35% at Wk 24 (assessed by MRI or CT).	SVR ≥ 35%: Momelotinib = 26.5%; Ruxolitinib = 29% (*p* = 0.011).	Grade 3 infections occurred in 7% of patients who received momelotinib and 3% of patients who received ruxolitinib.
SIMPLIFY-2 Phase III Trial	Adults with 2 or high-risk primary, post-PV or post-ET MF; currently or previously treated with ruxolitinib for ≥28 days and requiring transfusion or dose adjustment (*n* = 156). Patients with symptomatic intermediate-1 risk also included	Momelotinib 200 mg PO daily vs. BAT. The most frequent medications received by the patients in the BAT group were ruxolitinib, hydroxyurea, and corticosteroids. Some patients were treated with ruxolitinib plus additional therapies, most commonly hydroxyurea, followed by corticosteroids.	Primary endpoint: SVR ≥ 35% at Wk 24.	SVR ≥ 35% Momelotinib = 7%; BAT = 6% (*p* = 0.9).	Peripheral neuropathy occurred in 11 (11%) of 104 patients receiving momelotinib (one of which was grade 3) and in no patients in the BAT group.

Legend: BAT, best available therapy; ET, essential thrombocythemia; MF, myelofibrosis; PV, polycythemia vera; SVR, spleen volume reduction, BL, baseline; PD, progressive disease; TSS, total symptom score; DIPSS, Dynamic International Prognostic Scoring System; ITT, intention-to-treat; PMF, primary myelofibrosis; RBC-TD, red blood cell transfusion dependent; mTSS, modified total symptom score; PAC, pacritinib; RUX, ruxolitinib.

**Table 5:** Summary of trials investigating possible therapeutic approaches for disease progression or intolerance to initial therapy.

Study	Patients	Design	Endopoints	Results	Safety
JAKARTA-2 Single-arm, open-label, non-randomised, phase 2, multicenter study	Adult patients with diagnosis of intermediate or high-risk primary MF, post PV MF myelofibrosis, or post-essential thrombocythemia MF, found to be ruxolitinib resistant or intolerant	Oral fedratinib at a starting dose of 400 mg once per day, for six consecutive 28-day cycles.	Primary endpoint: spleen response (defined as the proportion of patients with a ≥35% reduction in spleen volume as determined by blinded CT and MRI at a central imaging laboratory)	Of 83 assessable patients, 46 (55%, 95% CI 44–66) achieved a spleen response.	Common grade 3–4 adverse events included anemia (37 [38%] of 97 patients) and thrombocytopenia (21 [22%] of 97), with 18 (19%) patients discontinuing due to adverse events.
PAC203 randomized dose-finding study	Adult patients with primary or secondary MF were eligible if they had intermediate-1, intermediate-2, or high-risk disease according to the DIPSS and were intolerant or failure ruxolitinib treatment.	165 patients were randomly assigned, and 161 received treatment with pacritinib 100 mg once per day (*n* = 52), 100 mg twice per day (*n* = 55), or 200 mg twice per day (*n* = 54)	Efficacy was based on SVR35 and ≥50% reduction in the 7-component TSS through week 24. Of 161 patients, 73% were intolerant of and 76% had become resistant to ruxolitinib; 50% met criteria for both.	SVR rates were highest with 200 mg twice per day, particularly among patients with baseline platelet counts < 50 × 10^3^/μL. Median percent reduction in TSS suggested a dose-response relationship (–3%, −16%, and −27%, respectively)	Common adverse events were gastrointestinal events, thrombocytopenia, and anemia.
PERSIST-2 Randomized phase III trial	Adults with int-1-, int-2-, or high-risk MF; platelets ≤ 100 × 10^9^/L; prior JAK2 inhibitors allowed (*n* = 311)	Patients were randomized 1:1:1 to pacritinib 400 mg once daily, pacritinib 200 mg twice daily, or BAT. The most commonly used active single agents in the BAT arm were ruxolitinib, hydroxyurea, and prednisone and/or prednisolone; some patients received watchful-waiting only	Primary endpoints: rates of patients achieving 35% or more SVR and 50% or more reduction in TSS at week 24.	Pacritinib (arms combined) was more effective than BAT for 35% or more SVR and had a nonsignificantly greater rate of 50% or more reduction in TSS. Pacritinib twice daily led to significant improvements in both end points over BAT.	For pacritinib once daily, pacritinib twice daily, and BAT, the most common (>10%) grade 3 or 4 adverse events were thrombocytopenia (32 patients [31%], 34 patients [32%], 18 patients [18%]), and anemia (28 patients [27%], 23 patients [22%], 14 patients [14%]).
MOMENTUM Double-blind, randomised, controlled, phase 3 study	Eligible patients were 18 years or older with a confirmed diagnosis of primary MF or post-PV or post-essential thrombocythemia MF	Patients were randomly assigned (2:1) to receive momelotinib (200 mg orally once per day) plus danazol placebo (i.e., the momelotinib group) or danazol (300 mg orally twice per day) plus momelotinib placebo (i.e., the danazol group), stratified by total symptom score (TSS; <22 vs. ≥22), spleen size (<12 cm vs. ≥12 cm), red blood cell or whole blood units transfused in the 8 weeks before randomisation (0 units vs. 1–4 units vs. ≥5 units), and study site.	Primary endpoint: MF Symptom Assessment Form (MFSAF) TSS response rate at week 24 (defined as ≥50% reduction in mean MFSAF TSS over the 28 days immediately before the end of week 24 compared with baseline).	A significantly greater proportion of patients in the momelotinib group reported a 50% or more reduction in TSS than in the danazol group (32 [25%] of 130 vs. six [9%] of 65; proportion difference 16% [95% CI 6–26], *p* = 0·0095).	The most frequent grade 3 or higher adverse events with momelotinib and danazol were anemia and thrombocytopenia. The most frequent non-haematological grade 3 or higher treatment-emergent adverse events with momelotinib and danazol were acute kidney injury and pneumonia

Legend: BL, baseline; DIPSS, Dynamic International Prognostic Scoring System; ECOG, Eastern Cooperative Oncology Group; IWG, International Working Group; JAKi, JAK2 inhibitor; MF, myelofibrosis; MFSAF, Myelofibrosis Symptom Assessment Form; PLT, platelets; PS, performance status; SVR, spleen volume reduction; SVR35, ≥35% spleen volume reduction; TSS, total symptom score, NAV, navitoclax; PBO, placebo; RUX, ruxolitinib; AE, adverse event; TSS50, ≥50% reduction in total symptom score; PV, polycythemia vera.
